# Advances in Sensing, Control and Path Planning for Robotic Systems

**DOI:** 10.3390/s26196253

**Published:** 2026-10-02

**Authors:** Rafal Szczepanski, Erfu Yang

**Affiliations:** 1 Institute of Engineering and Technology, Faculty of Physics Astronomy and Informatics, Nicolaus Copernicus University in Torun, ul. Wilenska 7, 87-100 Torun, Poland; 2 Robotics and Autonomous Systems Group, Department of Design, Manufacturing and Engineering Management, University of Strathclyde, Glasgow G1 1XJ, UK; erfu.yang@strath.ac.uk

## 1. Introduction

Robotic systems are increasingly expected to operate autonomously, safely, and efficiently in dynamic and uncertain environments [1,2,3,4,5]. To achieve these capabilities, the coordinated development of sensing [6,7,8], perception [9,10,11], state estimation [12,13,14], path and motion planning [15,16,17], and control [18,19,20] is required. Advances in a single component are often insufficient for real-world autonomy: sensory information must be transformed into meaningful representations of the environment, planning algorithms must generate feasible and safe motions, and control methods must ensure reliable execution on physical platforms. The need for integrated robotic solutions is particularly evident across application domains such as advanced manufacturing [21], logistics [22], agriculture [23], transportation [24], inspection [25], and oil & gas [26]. Within these domains, robots are increasingly deployed for tasks including service robotics [27,28,29,30,31], autonomous navigation [32,33,34,35,36], and multi-robot coordination [37,38,39,40,41], each demanding tightly integrated sensing, planning, and control. In these settings, robots may operate under incomplete or noisy measurements [42], inconsistent obstacle positions [43], kinematic and dynamic constraints [44], limited computational resources [45], and strict real-time requirements [46]. Consequently, current research is moving beyond isolated algorithmic improvements toward architectures that connect perception, decision making, planning, and control in a closed loop [47,48,49,50,51,52]. The Special Issue Advances in Sensing, Control and Path Planning for Robotic Systems was established to provide a venue for contributions addressing these interconnected research challenges. The nine papers published in this Special Issue cover a broad range of topics, including semantic-aware mobile robot navigation, kinematic sensing and estimation, collaborative swarm transport, sampling-based motion planning, learning from demonstration, object recognition and reconstruction, robot-joint characterization, trajectory generation for collaborative robots, and reinforcement learning for unmanned surface vehicles. Together, these contributions demonstrate that the development of autonomous robotic systems increasingly relies on the integration of heterogeneous sensing modalities, efficient data processing, planning methods that account for physical and operational constraints, and control strategies suitable for real-world execution. This Editorial provides a brief overview of the published works and highlights several directions for future research.

## 2. A Short Review of the Contributions in This Special Issue

The contributions in this Special Issue reflect the diversity of contemporary robotics research and illustrate the close relationship among sensing, control, learning, and planning. Abaza et al. [53] proposed a lightweight semantic-aware route planning framework for indoor mobile robots operating on resource-constrained edge hardware. Their approach combines monocular camera-based object detection with 2D LiDAR range estimation through an Angular Sector Fusion method. Semantic information is incorporated into the Nav2 Route Server using penalty-weighted graph routing, enabling online route replanning according to the detected characteristics of objects and obstacles. The study demonstrates the potential of computationally efficient sensor fusion and semantic routing for practical mobile robot navigation on physical hardware.

Bem et al. [54] presented an experimental validation of the direct kinematics of a passive Stewart-Gough platform with modified Cardan joints. Since the modified joint geometry invalidates the conventional spherical-joint assumptions used in standard hexapod models, the authors developed a numerical optimization-based framework for direct and inverse kinematics. Integrated absolute linear encoders provide autonomous pose sensing and make the platform suitable for reconfigurable and sensor-enabled industrial fixturing. This work emphasizes the importance of accurate sensing and model validation for parallel robotic mechanisms.

Nedjah et al. [55] addressed collaborative object transport in swarm robotics through a caging-based approach. The proposed method enables a group of robots to enclose and transport a cylindrical object while maintaining the formation required for stable collective manipulation. The use of state machines and PID controllers demonstrates how relatively simple control structures can support coordinated behavior in multi-robot systems. The contribution is relevant to distributed manipulation, cooperative transport, and collective robotic intelligence.

Dong et al. [56] introduced the FHQ-RRT*, an improved sampling-based path planning algorithm for mobile robots. The work aims to obtain high-quality, collision-free paths more efficiently than conventional RRT*-based methods. Sampling-based algorithms remain valuable for planning in complex state and configuration spaces. However, their practical application depends strongly on convergence speed, computational cost, and the quality of the resulting paths. The proposed improvement contributes to the continuing development of efficient global path-planning strategies for autonomous robots.

Learning from demonstration can enable robots to acquire task-relevant behaviors from human examples, but its performance may be limited by high-dimensional representations of demonstrations and trajectories. Manrique-Cordoba et al. [57] proposed an *N*-dimensional reduction algorithm for learning from demonstration path planning. The proposed dimensionality-reduction approach addresses this challenge by seeking compact representations that preserve information relevant to path generation. This direction is important for improving the scalability and usability of learning-based robot programming methods.

Zhang and Hashimoto [58] investigated garment recognition and reconstruction using object simultaneous localization and mapping. Their work combines two-dimensional instance segmentation with three-dimensional generative reconstruction based on signed distance functions. By creating dedicated datasets for garment image segmentation and synthetic three-dimensional mesh modeling, the authors address the difficult perception problem posed by deformable objects. The study is particularly relevant to the robotic manipulation of clothing and other flexible materials, where reliable visual understanding is a prerequisite for autonomous operation.

Turlapati et al. [59] studied the identification of intrinsic friction and torque ripple in a robotic joint equipped with integrated torque sensors. Accurate identification of these effects is essential for improving motion accuracy, force control, mechanical diagnosis, and model-based control performance. The application to wheel-bearing characterization further demonstrates the broader relevance of torque sensing and joint-level modeling for monitoring and maintenance-oriented robotic applications.

Szczepanski et al. [60] proposed an optimal path planning algorithm with built-in velocity profiling for a collaborative robot. The work considers not only the geometric properties of the planned path but also the velocity-related aspects that affect execution time, smoothness, and operational safety. This integrated perspective is valuable for collaborative robotic tasks in which the generated motion must be efficient while respecting the constraints of the robot and its workspace.

Finally, Dong et al. [61] reviewed reinforcement learning-driven autonomous path planning for unmanned surface vehicles. The paper discusses the current state of the field, its major challenges, and future prospects. Reinforcement learning provides a promising framework for adaptive decision making in complex and uncertain environments. However, practical deployment remains constrained by issues such as sample efficiency, safety, interpretability, training stability, and simulation to real world transfer. The review offers a useful perspective on the opportunities and limitations of reinforcement learning for maritime autonomous systems.

Overall, the papers published in this Special Issue show that advances in robotics increasingly emerge from the interaction of multiple research domains. Sensing supports environmental understanding and system perception. Planning transforms task objectives and environmental constraints into feasible robot behavior. Learning can improve adaptation and decision-making. And finally, control ensures that planned actions are executed reliably by physical systems.

## 3. Discussion and Future Directions

The papers published in this Special Issue highlight substantial progress in sensing, control, and path planning. At the same time, they reveal that the principal challenge in robotics is no longer solely the development of isolated algorithms. Instead, future progress will depend on the reliable integration of perception, estimation, planning, learning, and control into holistic systems. One important direction is the development of perception-aware and semantics-aware planning. Robots should not only detect obstacles but also interpret their properties, motion patterns, task relevance, and associated risks. Semantic information may influence route selection, velocity limits, safety margins, and the choice of manipulation or interaction strategy. The integration of vision-based perception, range sensing, semantic maps, and online replanning, as demonstrated in this Special Issue, is a promising step toward more context-aware robot behavior. A second direction concerns uncertainty-aware autonomy. Real robotic systems must deal with noise, imperfect calibration, model inaccuracies, communication delays, partial observability, and changing environments. Future planning and control methods should explicitly represent and manage these uncertainties. This includes robust state estimation, probabilistic environment representations, risk-sensitive planning, safe exploration, and controllers capable of maintaining stability and performance despite disturbances or modeling errors.

Beyond managing uncertainty, learning-based approaches will also remain a major area of research. Reinforcement learning, learning from demonstration, representation learning, and generative models can enable robots to adapt to complex tasks and unstructured environments. Nevertheless, their practical adoption requires improvements in sample efficiency, safety guarantees, interpretability, and generalization beyond training conditions. Hybrid methods that combine data-driven learning with model-based planning and control are particularly promising because they can exploit the flexibility of learned policies while retaining physical constraints, structure, and safety-related properties. Another relevant challenge is the transition from centralized single-robot systems to distributed multi-robot and human–robot environments. Swarm and cooperative robots require robust communication, decentralized coordination, formation maintenance, task allocation, collision avoidance, and resilience to individual robot failures. In parallel, collaborative robots must plan and execute motions that account for human presence, intentions, comfort, and safety. These requirements call for planning and control approaches that are not only technically efficient but also socially and operationally aware.

In addition to algorithmic and architectural advances, computational efficiency is equally important. Many robotic platforms operate with limited onboard processing power and strict real-time constraints. Future research should therefore consider resource-aware algorithm design, lightweight sensor fusion, edge computing, efficient optimization, adaptive model complexity, and hardware-aware implementations. Methods that can run reliably on embedded hardware while preserving safety and performance will be critical for the widespread deployment of autonomous robots. Finally, more experimental validation and reproducibility are needed to bridge the gap between simulation studies and real-world deployment. Open datasets, open-source software, standardized benchmarks, and systematic hardware experiments can help researchers evaluate generalization, robustness, computational requirements, and safety. Evaluation criteria should also extend beyond path length and execution time to include energy consumption, tracking accuracy, computational latency, resilience to disturbances, interaction safety, and long-term system reliability.

In conclusion, the nine papers in this Special Issue provide a representative overview of recent advances in sensing, control, learning, and path planning for robotic systems. They demonstrate the importance of integrating environmental perception, system-level sensing, motion generation, adaptive decision making, and reliable control. We hope that this collection will stimulate further interdisciplinary research toward robotic systems that are more intelligent, autonomous, efficient, robust, and safe in real-world operating conditions.
